# Nanotechnology-Based Celastrol Formulations and Their Therapeutic Applications

**DOI:** 10.3389/fphar.2021.673209

**Published:** 2021-06-11

**Authors:** Pushkaraj Rajendra Wagh, Preshita Desai, Sunil Prabhu, Jeffrey Wang

**Affiliations:** Department of Pharmaceutical Sciences, College of Pharmacy, Western University of Health Sciences, Pomona, CA, United States

**Keywords:** celastrol, nanoformulations, targeting, bioavailability, anti-inflammatory, anti-autoimmune, anti-cancer, anti-oxidant

## Abstract

Celastrol (also called tripterine) is a quinone methide triterpene isolated from the root extract of *Tripterygium wilfordii* (thunder god vine in traditional Chinese medicine). Over the past two decades, celastrol has gained wide attention as a potent anti-inflammatory, anti-autoimmune, anti-cancer, anti-oxidant, and neuroprotective agent. However, its clinical translation is very challenging due to its lower aqueous solubility, poor oral bioavailability, and high organ toxicity. To deal with these issues, various formulation strategies have been investigated to augment the overall celastrol efficacy *in vivo* by attempting to increase the bioavailability and/or reduce the toxicity. Among these, nanotechnology-based celastrol formulations are most widely explored by pharmaceutical scientists worldwide. Based on the survey of literature over the past 15 years, this mini-review is aimed at summarizing a multitude of celastrol nanoformulations that have been developed and tested for various therapeutic applications. In addition, the review highlights the unmet need in the clinical translation of celastrol nanoformulations and the path forward.

## Introduction

Clinical translation of bioactive compounds extracted from medicinal plants has gained substantial interest over the past several years due to their superior pharmacological activities especially as anti-inflammatory, anti-tumor, and neuroprotective agents. One such widely investigated medicinal plant is *Tripterygium wilfordii*, a perennial vine of the Celastraceae family, commonly known as “thunder god vine” or “lei gong teng.” It is used traditionally in China to treat autoimmune disorders such as rheumatoid arthritis, Crohn’s disease, and type 1 diabetes (Cascão et al., 2017). The plant is rich in phytochemicals that comprise triterpenoids and alkaloids, which are mainly extracted from the root pulp of the plant. Among these phytochemicals, the most abundant and promising bioactive compound is celastrol.

**FIGURE 1:**
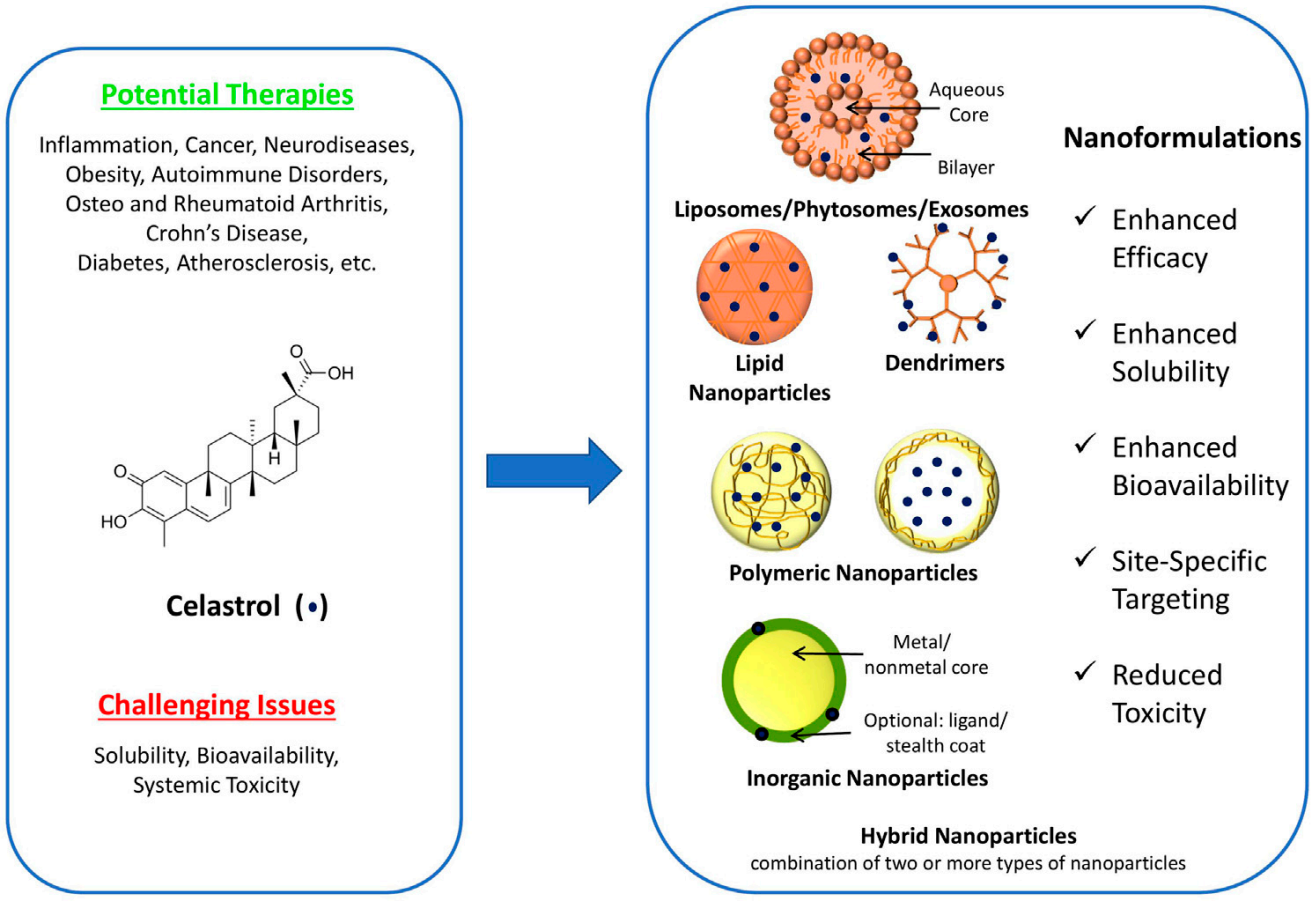
Structure of celastrol, potential therapies, challenging issues, and schematic representation of various celastrol nanoformulations [modified from Desai et al. (2012)].

Celastrol, also known as tripterine, is a quinone methide triterpene (Figure 1). It has gained importance over the past two decades due to its potent anti-inflammatory (Pinna et al., 2004; Shaker et al., 2014; Ma et al., 2015), anti-cancer [gastric and ovarian cancers (Xu et al., 2019; Chen et al., 2020), cervical cancer (Zhou et al., 2017), and hepatocellular carcinoma (Wang et al., 2019; Chen et al., 2020; Du et al., 2020)], neuroprotective (Paris et al., 2010; Li et al., 2012; Jiang et al., 2018), and anti-oxidant (Cleren et al., 2005) activities. However, albeit potent, its clinical translation is impeded due to two main disadvantages that are poor water solubility of 0.044 mg/ml at 25°C (BCS class IV drug) (Yang et al., 2019), which limits its bioavailability, and high systemic toxicity resulting from its narrow therapeutic index (Zhang et al., 2014; Shi et al., 2020).

The reported therapeutic dose of celastrol against various mouse models is in the range of 3–5 mg/kg (Yang et al., 2006). At these doses, though effective, systemic toxicities including cardiotoxicity (Liu et al., 2019), hepatotoxicity (Jin et al., 2019), and nephrotoxicity (Wu et al., 2018) have been reported, whereas lower doses, though safe, show limited efficacy. To overcome the toxicity issues while achieving the desired therapeutic efficacy, various drug delivery approaches have been investigated that include combination with other chemotherapeutic agents such as afatinib, axitinib, and gefitinib (Zhang et al., 2014; Choi et al., 2016; Gao et al., 2019; Lee et al., 2019; Zhao et al., 2019; Dai et al., 2020), combination with traditional Chinese medicines such as betulinic and ellagic acids (An et al., 2015; Duan et al., 2019), overcoming multidrug resistance (Metselaar et al., 2019), nanoparticulate drug delivery systems (Qi et al., 2014; Hakala et al., 2020; Li et al., 2020), and combination with nucleic acid (Huang et al., 2017). Among these, nanoparticulate drug delivery systems/nanoformulations of celastrol have been widely reported as a promising strategy to effectively deliver drug at the target site rendering enhanced efficacy and safety.

Furthermore, celastrol is classified as a BCS class IV molecule (exhibiting low solubility and permeability), and therefore, solubility and permeability enhancement strategies are the most effective in improving the bioavailability of the drug. In this context, nanoformulations, owing to its smaller size and targeting potential, offer advantages of enhanced solubility (high surface-to-volume ratio) and permeability, both of which are advantageous parameters in enhancing the bioavailability of celastrol. Thus, in view of a multitude of advantages offered by the nanotechnology, celastrol nanoformulations have been widely explored and reported in the literature. Specifically, celastrol nanoformulations have shown significant benefits in several therapeutic applications against prostate cancer, breast and pancreatic cancers, non-small-cell lung cancer, ovarian cancer, and human colon cancer and other applications in treating rheumatoid arthritis, polycystic kidney disease, inflammation, and Parkinson’s disease (schematically depicted in Figure 1) (Abbas et al., 2007; Salminen et al., 2010; Yadav et al., 2010; Mou et al., 2011; Kim et al., 2013; Chang et al., 2018; Qi et al., 2018; Zha et al., 2018; Lin et al., 2019; Song et al., 2019; Wang et al., 2019; Yan et al., 2020). This review summarizes such state-of-the-art therapeutic applications of celastrol nanoformulations in the subsequent sections. For this, peer-reviewed publications over the past 15 years in the area of celastrol nanoformulations were searched, categorized based on the therapeutic application, and summarized to develop the comprehensive mini-review as presented.

## Celastrol Nanoformulations and Their Therapeutic Applications

### Cancer

NF-κB inhibition is the most commonly reported pharmacological mechanism of celastrol’s anti-cancer activity (Elhasany et al., 2020). Celastrol also inhibits M2-like polarized tumor-associated macrophages that are involved in tumor metastasis. In an *in vivo* study (Yang et al., 2018), the expression of M2-like genes by quantitative real-time PCR showed that genes including MRC1, Arg1, Fizz1, Mgl2, and CD11c were up-regulated by IL-13 administration, which was greatly reduced by celastrol co-administration. Other molecular targets include liver X receptor α and ATP-binding cassette transporter A1 (Zhang et al., 2020), microRNA-21 (Yao et al., 2019), androgen receptor/microRNA-101 (Guo et al., 2015), lipoprotein receptor-1 (Gu et al., 2013), microRNA-33a-5p/E2F7 transcription factor (Liu et al., 2020), PI3K–Akt–mTOR signaling (Li et al., 2018), mitochondrial ubiquitination (Hu et al., 2017), CXC chemokine receptor type 4 (Yadav et al., 2010), peroxisome proliferator–activated receptor α signaling (Zhao et al., 2020), and transforming growth factor β1 (Kang et al., 2013). In spite of multi-target anti-cancer potency of celastrol, its clinical translation has not been realized due to poor bioavailability, inadequate tumor targeting, and high toxicity. The nanoformulations developed and investigated to overcome these challenges for various cancers are described below, and additional nanoformulations not discussed in detail are summarized in Table 1.

**TABLE 1:** Literature overview of nanotechnology-based celastrol formulations.

Nanocarrier type	Indication	Key outcomes	Reference
**Polymeric**			
Silk fibroin nanoparticles	Cancer	Size: ∼ 300 nm	Onyeabor et al. (2019)
		2.4-fold bioavailability enhancement *in vivo*	
Micelles	Macrophage-induced corneal neovascularization (CNV)	Size: ∼ 48 nm	Li et al. (2012), Li et al. (2016)
		Suppressed macrophage-induced CNV *in vitro* and *in vivo*	
		Modulation of MAPK and NF-κB signaling pathways	
Micelles	Retinoblastoma	Size: ∼ 48 nm	Li et al. (2012)
		Growth inhibition in the mouse xenograft model by inhibition of NF-κB	
		Downregulation of expression of Bcl-2 leading to apoptosis	
Micelles	Cancer	Size: 86.8 ± 7.6 nm	Tan et al. (2018)
		Internalization of micelles in mitochondria *in vitro* and *in vivo*	
		Modulation of mitochondria-mediated apoptotic pathway by increasing ROS levels	
Micelles	Rheumatoid arthritis	Regulation of the NF-κB and Notch1 pathways	An et al. (2020)
		Relieved main rheumatoid arthritis symptoms (articular scores, ankle thickness, synovial inflammation, bone erosion, cartilage degradation)	
Micelles	Atherosclerosis, inflammation	Size: 14.8–17.9 nm (size increased with increase in drug loading)	Allen et al. (2019)
		Reduced TNF-α secretion, number of neutrophils, and inflammatory monocytes within atherosclerotic plaques	
		Inhibition of NF-κB signaling pathway	
Nanoconjugates	Cancer	Internalization of nanoparticles in MCF-7 and suppression of tumor growth *in vitro* and *in vivo*	Abdelmoneem et al., (2021)
		Inhibition of NF-κB, TNF-α, COX-2, and Ki-67	
Nanoparticles	Prostate cancer	Size: 189.1 ± 2.9 nm	Sanna et al. (2015)
		Suppressed proliferation, angiogenesis, and cell cycle protein markers in PC3 cell line *in vitro*	
		Significant decrease in the expression of Ki-67, PCNA, TNF-R1/2, and Fas, as well as induction of p21 and p27	
Nanoparticles	Prostate cancer	Size: 75.4 nm	Yin et al. (2017)
		Enhancement in anti-tumor effect *in vivo*	
		Enhanced efficacy by pre-saturation of reticuloendothelial system by the blank nanoparticles	
**Lipid**			
Microemulsion	Ovarian cancer	Size: ∼50 nm	Zhao et al. (2020)
		Combinational and tumor-targeted cancer therapy	
		Active tumor targeting via transferrin and cell-penetrating peptide	
		Reduced the toxicity of tripterine against the liver and kidney	
		Enhanced antitumor efficacy *in vivo*	
Microemulsion	Lung cancer	Size: 69.2 ± 3.3 nm	Zhang et al. (2019)
		Combination treatment of nano-*ß*-elemene and celastrol showed synergistic anti-cancer efficacy *in vivo*	
		No obvious systemic toxicity *in vivo*	
Liposome	Lung cancer	Size: 89.61 ± 0.53 nm	Song et al. (2011)
		Enhanced permeability in four-site perfusion rat intestinal model due to cell membrane–mimicking liposome	
		Enhanced anti-tumor activity *in vivo*	
Nanostructured lipid carrier	Enhanced absorption	Size: 109.6 ± 5.8 nm	Zhou et al. (2012)
		Delayed drug release profile with enhanced absorption in rat intestinal perfusion model	
Nanostructured lipid carrier gel	Arthritis and inflammation	Size: 26.92 ± 0.62 nm	Kang et al. (2018)
		Combination of celastrol and indomethacin lipid nanocarriers showed significant reduction in paw edema model *in vivo*	
		Inhibition of inflammation and pain by modulating IL-1β, TNF*-α, ß-*endorphin, and substance *p*	
**Inorganic**			
Gold nanourchins	Glioblastoma	Significant reduction in the pro-survival signaling via the PI3 kinase–Akt pathway	Maysinger et al. (2018)
		Significant inhibition of glioblastoma cells	

Cancer tumorigenesis and metastasis is induced by multiple mechanisms including migration, invasion, and angiogenesis. The tumor microenvironment (TME) plays a key role in these carcinogenic mechanisms, and multiple strategies have been investigated to alter the TME in order to treat cancers. Among various pharmacological responses, celastrol is reported to inhibit NLRP3 inflammasome, which in turn impedes the macrophage potency to promote migration and invasion of melanoma cells (Lee et al., 2019). To effectively deliver celastrol for treatment of melanoma, self-assembling amphiphilic polymer/celastrol prodrug nanoparticles were developed by chemically conjugating celastrol to the diblock polymer methoxy-poly(ethylene glycol)-*b*-poly(L-lysine) (Li et al., 2020). This celastrol prodrug underwent self-assembly to form stable micellar nanoparticles (103.1 ± 10.7 nm) due to hydrophobic and electrostatic interactions between the drug and the polymer. An *in vivo* study in the B16F10 mouse melanoma model showed significant uptake of the nanoparticle formulation due to the enhanced permeability and retention (EPR) effect that resulted in tumor growth reduction and lowered toxicity compared to that of celastrol alone, confirming the potential of functionalized nanoparticle-mediated drug targeting as a safe and effective tool (Li et al., 2020). In another investigation, a celastrol nanoemulsion was reported to downregulate programmed cell death-ligand 1, eliciting strong immunogenic cell death in a bilateral tumor model. This can be viewed as a promising avenue of chemotherapy-induced cancer immunotherapy (Qiu et al., 2021). Celastrol was also reported to have inhibitory effect on tumor-associated fibroblasts that play a critical role in desmoplastic melanoma. In view of the strong anti-fibroblast and immunomodulatory effects of celastrol, it has also been combined with potent anti-cancer drugs to achieve simultaneous chemo-immunotherapy in melanoma treatment. For instance, Liu et al. developed TME-responsive targeted aminoethylanisamide polymeric nanoparticles comprising a drug combination of the anti-cancer agent mitoxantrone and celastrol in a 5:1 ratio. The nanoparticles exhibited a size of 112 ± 6 nm with > 75% drug encapsulation for both drugs. *In vivo* melanoma tumor model studies confirmed inhibition of cancer progression/metastasis and TME immunosuppression, confirming the hypothesis synergistic anti-cancer efficacy with combination drug nanoparticles of mitoxantrone and celastrol (Liu et al., 2018). Similarly, bio-mimicking polyethylene glycol–poly(lactic-co-glycolic acid) (PEG–PLGA) nanoparticles coated with the neutrophil membrane showed higher internalization and apoptosis in the murine melanoma B16F10 cell line as compared to uncoated nanoparticles. This coating also helped to increase the biodistribution in the tumor xenograft model (Zhou et al., 2019).

Celastrol has also been reported to have potent activity against ovarian cancer via mechanisms that include intracellular accumulation of reactive oxygen species (ROS), apoptosis, cell cycle arrest (G2/M phase), and ultimately cell growth inhibition (Xu et al., 2019). For instance, an *in vitro* study with the ovarian cancer SKOV3 cell line showed proportional increase in intracellular ROS concentration with increased exposure to celastrol, confirming the ROS responsiveness of celastrol (Xu et al., 2019). To enhance the clinical efficacy with reduction in toxicity, various nanoformulations have been investigated. Furthermore, to enhance ovarian cancer targeting, Niu et al. prepared celastrol nanoparticles using the poly(lactic-*co*-glycolic acid)–poly(ethylene glycol) methyl ether (PLGA–mPEG) polymer and coated them with folic acid for active tumor targeting. Folate receptors upregulate in tumor tissue, and hence, the use of folic acid–coated nanoparticles enables enhanced uptake via active targeting. The prepared nanoparticles displayed the encapsulation efficiency of 95% with a particle size of 155 nm and showed significant enhancement in ROS levels’ inhibitory potential against SKOV3 cells with prolonged treatment time (Niu et al., 2020). The folate receptor tumor targeting approach was also investigated by Law et al. wherein they developed folic acid–functionalized celastrol-conjugated gold–polymer nanoparticles to achieve active targeting against breast cancer. The developed nanoparticles showed significant enhancement in apoptosis in 2D and 3D breast tumor models compared to celastrol alone. The nanoparticles also exhibited higher cellular uptake efficiency and lower colony-forming assay units, confirming enhanced uptake of the nanoparticles leading to improved efficacy (Law et al., 2020). Celastrol nanosuspension with a size of 147.9 nm has also been developed and investigated for breast cancer treatment. Celastrol was stabilized in an amorphous form in the nanosuspension, hence enhancing its dissolution significantly to 69.2% in 48 h. Compared to intravenous injection of the anticancer drug paclitaxel, the oral and intravenous treatment with celastrol nanosuspension showed similar and higher tumor inhibition rates, respectively. Hence, the unique property of nanoformulations to enhance dissolution of poorly soluble drugs such as celastrol can be explored to enhance solubility and in turn the *in vivo* efficacy (Huang et al., 2020).

Non-small-cell lung cancer is the most predominant lung carcinoma, and tyrosine kinase inhibitors (TKIs) are classically used for chemotherapy. Celastrol has gained particular attention in treatment of this type of cancer due to its serine threonine protein kinase (Akt) inhibitory potential, which is proven to be very effective if combined with TKIs. Particularly, Xie et al. developed a nano-product comprising the TKI gefitinib and celastrol along with a fluorescent diagnostic probe. The combination nano-prodrug approach not only allowed fluorescence and optoacoustic tumor imaging but was also proven to be superior, exhibiting significant tumor inhibition in an orthotopic mouse tumor model (Xie et al., 2020). In another study (Zhao et al., 2018), a nanoformulation comprising celastrol-loaded glucolipid-like conjugates tagged with avb3-ligand tetraiodothyroacetic acid was developed to inhibit the NF-kB signaling pathway in lung and breast metastatic cancer cells. The targeted nanoformulation was selectively taken up by the cells via the avb3 receptor–mediated interaction. The study showed reduction in the apoptotic marker MMP-9 *in vivo*, confirming that the prepared celastrol-loaded micelles suppressed breast tumor invasion and lung metastasis. In addition, self-assembled micelles containing covalently conjugated celastrol–PEG–ginsenoside Rh2 were developed for endosomal/lysosomal delivery. The formulation showed significant enhancement in the bioavailability due to introduction of PEG that imparted stealth (long circulation) properties to the nanoparticles and showed synergistic anti–lung cancer activity due to the combination approach (Li et al., 2017).

In addition to the use of folic acid for active tumor targeting, multiple other active targeting strategies have also been reported in this area. For instance, glucose was used as an affinity ligand to decorate mesoporous silica nanoparticles for the delivery of celastrol with high specificity to HeLa and A549 cancer cells. To further increase the specificity, poly(ethylene imine) was surface-branched on the nanoparticles that increased the overall positive charge and hence the cellular uptake (Niemelä et al., 2015). In another interesting study, theranostic (combining therapeutics with diagnostics) nanoparticles incorporated with a drug combination of celastrol and sulfasalazine were developed for targeted breast cancer management. Specifically, SPION-tagged amphiphilic zein–chondroitin sulfate micelles were used to achieve simultaneous CD44–tumor-targeted drug delivery of celastrol and sulfasalazine along with MRI. The combination nanoplatform showed highest efficacy compared to non-targeted and free drug treatment groups confirming its superiority (Elhasany et al., 2020). Furthermore, folate receptor–targeted liposomes carrying combination of celastrol and irinotecan have also been reported for lung and breast cancer treatment (Soe et al., 2018). The liposomes exhibited improved cellular uptake and apoptosis when tested in multiple cancer cell lines (MCF-7, MDA-MB-231, A549). The *in vivo* studies in MDA-MB-231 tumor-bearing female BALB/c nude mice confirmed highest suppression with the liposomal treatment group (Soe et al., 2018). Another vesicular nanoformulation investigated for lung cancer treatment was celastrol exosomes that showed efficacy against A549 and H1299 lung cancer cells. Additionally, when tested in a xenograft model, exosomal celastrol presented enhanced anti-tumor efficacy compared to free celastrol and was devoid of kidney and liver toxicity, confirming its promise in lung cancer treatment (Aqil et al., 2016).

Celastrol has also been proven to be effective against prostate cancer. For example, investigation of celastrol poly(ε-caprolactone) nanoparticles against prostate cancer cell lines (LNCaP, DU-145, and PC3) revealed significant inhibition (IC_50_ < 2 µM) with modulation of apoptotic proteins (Sanna et al., 2015). In another study, polycaprolactone polymeric tripterine nanoparticles were prepared with a size of about 75 nm. The nanoparticles were proven to elicit significant tumor reduction compared to the free drug in LNCaP cell BALB/c mice xenograft model (Yin et al., 2017). Lipid nanocarriers have also been investigated for this purpose. For example, Chen et al. developed nanostructured lipid carriers (NLCs) of celastrol and coated them with the cell-penetrating peptide to achieve active tumor targeting. The NLCs (size: 126.7 ± 9.2 nm) showed a controlled drug release profile with enhance absorption *in vivo* due to NLCs’ colloidal form and nanosize. Specifically, the NLC formulation showed 484.75% enhancement in bioavailability compared to a plain drug (Chen et al., 2012). The similar group further investigated the efficacy of the targeted NLCs in the prostate cancer model *in vivo*. The studies confirmed enhanced anti-tumor effect and reduction in tumor markers (necrosis factor-alpha, interleukin-6 cytokine) compared to plain drug control in a dose-dependent manner (Yuan et al., 2013).

Celastrol has also been proven effective against pancreatic carcinoma (Cao et al., 2019). Celastrol-loaded neutrophil-mimicking nanoparticles were demonstrated to achieve pancreas-specific drug delivery by overcoming the blood–pancreas barrier *in vivo*. For this, the poly(ethylene glycol) methyl ether-block-poly(lactic-co-glycolic acid) polymer was used as a naive neutrophil membrane coating. The coating induced neutrophil-like properties to the nanoparticles that enhanced their uptake by the cells both *in vitro* and *in vivo. In vitro* evaluation of these nanoparticles in the lipopolysaccharide-stimulated RAW264.7 macrophages and L929 cells showed marked cellular uptake and internalization. Furthermore, the *in vivo* anti-tumor efficacy study in the female pancreatic cancer mice model proved enhanced and site-specific anti-tumor activity. Similarly, celastrol-loaded silk fibroin (SF) nanoparticles were synthesized and studied in human pancreatic cancer cells (MIA PaCa-2 and PANC-1). SF nanoparticles showed lower IC_50_ values in both the cell lines compared to free celastrol (Ding et al., 2017).

To target celastrol for solid tumor treatment, mesoporous silica nanoparticles capped with PEGylated polyaminoacid were prepared for mitochondria-targeted delivery of celastrol. The targeted nanoparticles were shown to have enhanced efficacy in the SCC-7 cancer cell–bearing xenograft tumor mice model (Choi et al., 2018). In a study to examine apoptotic effects of celastrol on cancer cells, SW620 colorectal cancer cells both *in vitro* and in nude mice were conducted along with biosafety studies in zebrafish and xenograft mice models. The prepared dendrimer bioconjugate of celastrol showed a particle size of 40 nm (spherical in shape) and induced apoptosis in the colorectal cancer cells *in vitro* and in mice with reduction in local and systemic toxicity (Ge et al., 2020). To summarize, a plethora of celastrol nanoformulations have shown their potential to enhance the efficacy and safety profile of celastrol in cancer treatment.

### Osteoarthritis and Rheumatoid Arthritis

Celastrol is a potent therapeutic agent investigated for the treatment of osteoarthritis and rheumatoid arthritis. It elicits treatment benefit by regulating functions of Th1 and Th2 cells, fibroblasts, macrophages, and endothelial cells that play critical roles in the etiology and pathogenesis of arthritis. In addition, celastrol inhibits numerous inflammatory chemokines that include mundane T cells expressed and secreted, monocyte chemoattractant protein 1, macrophage inflammatory proteins, and growth-regulated oncogene/keratinocyte chemoattractant. In addition to these molecular targets, celastrol also modulates the function of metalloprotein, JNK and MEK1 pathways (Song et al., 2019). Celastrol nanoformulations have been reported to further enhance the anti-inflammatory efficacy while offering promising safety profile. For example, celastrol-loaded palmitic acid–modified bovine serum albumin (PAB) nanoparticles and bovine serum albumin (BSA) nanoparticles were developed and tested for anti-inflammatory response in the AIA rats for scavenger receptor-A targeting via intravenous injection treatment (Gong et al., 2020). The celastrol PAB nanoparticles significantly improved rheumatoid arthritis symptoms at a lower dose with fewer toxic effects compared to the celastrol BSA nanoparticles. Furthermore, mechanistically, celastrol PAB nanoparticles were proven to enhance scavenger receptor-A targeting due to high electronegativity (excipients: BSA and palmitic acid) compared to celastrol BSA nanoparticles (excipient: BSA) (Gong et al., 2020). In another study, phytosomes with a combination of celastrol and selenium were administered via oral gavage to treat arthritis in male AIA rats. These phytosomes enhanced the transepithelial transport of drugs due to smaller phytosomal size (126 nm) and enhanced nanoparticle transmembrane diffusion. This enhanced uptake resulted in significant alleviation of the arthritis symptoms and also lowered the inflammatory factors (Zhu et al., 2020).

### Miscellaneous

Celastrol nanoformulations have been studied to benefit in treatment of obesity (Liu et al., 2015), diabetes, lipid accumulation, psoriasis (Zhou et al., 2011), etc. For example, celastrol-loaded polyethylene glycol–polycaprolactone nanomicelles effectively ameliorated body weight, lipid accumulation, diabetes, and metabolic dysfunction in diet-induced obese mice. Furthermore, histopathological examination of the high-fat-diet–induced obese mice model confirmed that the treatment with celastrol nanoformulation did not result in any anal irritation or intestinal disturbance otherwise seen in control or plain celastrol–treated animals. Hence, celastrol nanomicelles can be deemed more effective and safer (Zhao et al., 2019). Celastrol has also been proven to be effective in treatment of renal diseases (Tang et al., 2018). But its severe systemic toxicity limits its use. Guo et al. prepared mesangial cell–targeting celastrol nanoparticles using human serum albumin for ameliorating the effects of mesangioproliferative glomerulonephritis (MsPGN). They confirmed the selective uptake of celastrol nanoparticles via *in vivo* fluorescence imaging and semiquantitative fluorescence intensity measurement of the kidneys (excised 5 min after tail vein injection of nanoparticles), and the nanoparticles with size 95 nm showed maximum uptake by the kidneys (Guo et al., 2017). The *in vivo* evaluation clearly showed that the formulation not only reduced the systemic toxicity but also minimized the off-targeting effects of celastrol. It also showed potent effects against proteinuria, inflammation, glomerular hypercellularity, and extracellular matrix (ECM) deposition in an anti-Thy1.1 nephritis rat model. This was attributed to the anti-inflammatory, anti-proliferative, and anti-fibrotic mechanisms, highlighting celastrol as a promising agent for the treatment of MsPGN such as IgA nephropathy (Guo et al., 2017). Multiple studies as described earlier have shown that nanoparticles enhance the bioavailability of celastrol. In one such specific study, Freag et al. encapsulated celastrol in a self-assembled phospholipid-based phytosomal nanocarrier system. The *in vivo* pharmacokinetic data in rabbits indicated that the phytosomes increased the bioavailability and C_max_ by fourfold and fivefold, respectively, compared to the celastrol suspension. The authors attributed the bioavailability enhancement to meticulous use of phospholipids that not only retain cell membrane fluidity but also potentially enhance the rate and extent of intestinal drug absorption and enhancement in the aqueous solubility of celastrol by incorporating it in a nanoformulation (Freag et al., 2018).

## Conclusion and Future Prospects

Medicinal plants containing bioactive constituents are a great resource for modern drug development, and *Tripterygium wilfordii* is one of them. Its major constituent celastrol has numerous pharmacological actions including anti-inflammatory, anti-obesity, anti-diabetic, and anti-cancer activities (Cascão et al., 2017; Chen et al., 2018; Hou et al., 2020; Yan et al., 2020; Lu et al., 2021). However, there are multiple challenges in translating traditional herbal medicines and their active constituents to modern drug therapies. In particular, celastrol presents issues of low solubility, bioavailability, and toxicity (Zha et al., 2018). For seven decades, numerous attempts have been made to overcome the problems of celastrol delivery, and recently, nanotechnology-based formulations have shown great promise in enhancing its overall pharmacological efficacy and safety. Celastrol nanoformulations (enhanced permeation, retention, tumor targeting, and controlled drug release) can be looked upon as a promising avenue toward successful clinical translation of this potent bioactive agent toward treatment of various human diseases (Fang and Tang, 2020). More importantly, the universal challenge of clinical translation of nanomedicine needs more attention, and as rightly pointed out by Sun et al. (2020), pharmaceutical scientists, engineers, chemists, and material scientists must work in synergy to develop stable, scalable, and effective nanoformulations. Furthermore, regulatory authorities worldwide are developing specific guidelines to streamline the approval of nanomedicine-based products that would help in successful clinical translation of these formulations in the near future (Paradise, 2019).
